# The Home Clinic *or* All in a Day's Work of Dr. Fics

**DOI:** 10.1111/1751-7915.13520

**Published:** 2019-12-19

**Authors:** Kenneth Timmis

**Affiliations:** ^1^ Institute of Microbiology Technical University of Braunschweig Braunschweig Germany

(*St. Albans, U.K., 7.15, 21st of March, 2021: Maisy Collins clears off the last crumbs of toast from the dining table, opens her laptop, clicks the NCIC* * *link on the toolbar of her browser, enters her unique Corset* † *identifier, and clicks the medical consultation option*.)

*Fics*:‡Good morning Maisy – how are you? You are certainly looking well.

*Maisy*: Good morning Dr. Fics – yes, I am fine, thank you. How are you?

*Fics*: Very well, thank you, apart from the dreadful weather. I see you have rain again in St. Albans and, with this cold wind from the North, it will not be terribly pleasant for you to take your poodle for walkies.

*Maisy*: Yes, but you know that the British, and especially their beloved dogs, are used to this type of weather. Not all retirees migrate to Madeira for Winter!

*Fics*: Okay, Maisy: what would you like to discuss today?

*Maisy*: Well, I received on Friday an e‐mail from the Driver and Vehicle Licensing Agency informing me that, as I have reached the age of 70, I need to renew my driving license. Apparently, it has automatically received from you all my health‐relevant info, except for an eyesight test not older than 3 months, and informed me that I must take this online.

*Fics*: Right! Just scanning your file in the *Patient Data Centre* * – oh, great: you have a *super smart* 5G Phone with dual high definition 3‐D cameras, projector and precision laser distance meter. I assume you already have it linked to and synched with your computer?

*Maisy*: Yes, Doctor.

*Fics*: OK! Now what we need for this is a blank wall, 3‐5 feet away from your chair and table, and your ssPhone in its stand just in front of you and pointing to the wall. Can you configure this for me, and click the “share my computer control with *NCIC*” option? And we need a clean cloth or towel.

*Maisy, 1 minute later*: Everything done!

*Fics*: Okay: now I see you well and the wall. The angle is not exactly what I need: could you please rotate the phone slightly to the left…..stop ‐ is now perfect! Okay: are you sitting comfortably? Please cover your left eye with your cloth. I am now going to operate the ssPhone projector: do you see the letters and numbers on the wall?

*Maisy*: Yes, Doctor.

*Fics*: Okay: please read the bottom row for me

*Maisy*: S, 3, 8, W, N, Z

*Fics*: Just perfect! Now you see a grid of smaller letters: please read the middle row for me

*Maisy*: M, A, 2, Q, 4

*Fics*: Again perfect! Now please read the upper row of the new display.

*Maisy*: oohhh: this gets really difficult: 7…I think the next is a U….maybe a Y, a 6? Sorry, I cannot read the last one.

*Fics*: Very good! Now please cover your right eye and we'll repeat the exercise. Could you please cover a larger area around your eye with the cloth…….Thank you.

*Maisy*: 9, 5, O, P, B; S, G……4…….3…. K; I think the next is L then F… no, sorry, I am just guessing.

*Fics*: No problem, Maisy: you have rather average eyesight for your age. Obviously, your left eye is weaker than your right, but is still adequate for driving at the moment. The DVLA now has the result and will be issuing your new driving license this morning.

*Maisy*: Thank you Doctor, that was so efficient!

*Fics*: On the other hand, you do have developing vision issues that need attention, so I would recommend an ophthalmological assessment. May I make an appointment for you at your local hospital?

*Maisy (somewhat deflated)*: Oh, well if you think it is necessary….

*Fics*: Okay: I have made an appointment for you on the 23rd, the day after tomorrow, at in the Ophthalmology Clinic at St. Albans Infirmary with a Dr. O'Culah.^3^ Well, if there is nothing else, I wish you a lovely day, despite the weather.

*Maisy*: Thank you Doctor; I wish you the same.

*(Estepona, Spain, 7.37, 21st of March, 2021: Tristram Waldringham puts his sonic toothbrush back in its holder, walks to the balcony overlooking the sea, sits down and clicks the NCIC link of the browser of his computer, enters his unique Corset identifier, and clicks the medical consultation option.)*

*Fics*: Good morning Tris: how are you this beautiful morning on the Costa del Sol?

*Tris*: Good morning Doctor. I am fine, thank you and, yes, we do have lovely weather at the mo.

*Fics*: From the info in the *Patient Data Centre,* *. I see that you have received an automated reminder to arrange a repeat digital dermoscopy. Is this the reason for your contact this morning?

*Tris*: Yes, Doctor: it is time for my annual “mole hunt”.

*Fics*: Okay: I will order a scan frame for you; ah, yes, there is one available 2 km away. I'll get *MamaSon* § to deliver it straight away ‐ one moment – okay: you should be receiving it in about 10 min, so we can stay online while we check a few things in advance. Firstly, please switch on and link your ssPhone to your computer…..Excellent! Now as you will remember from previous dermoscopies, we need to place the scan frame against a wall, near a power socket, with plenty of space in front for you to move around, so please use the phone to show me the wall you have selected. Aahh: yes, that location will be perfect. Now, have you noticed any change in your moles: appearance, enlargement, darkening?

*Tris*: No, Doctor, though of course, there are bits of my anatomy that are not easy for me personally to monitor. One moment, there is someone at the door, probably *MamaSon's* delivery of the frame…….Ah, yes, here it is: the nice lady is just setting it up and plugging it in.

*Fics*: Very good: I can now see you from the high definition camera on the dermoscopy frame: I'll just check that it moves smoothly on its tracks– perfect! Okay, please remove all garments and position yourself in front of the frame……excellent…please lift your head a little…very good. Okay, please keep perfectly still ‐ I am now starting the scan. Very nice: now please make a quarter turn to the left…….Great: now lift your right arm…quite still….very good. Now another quarter, arm down….good, now another quarter…..now left arm up…excellent! You have a couple more naevi…..we will now take some close‐ups of those, and several others to see if there have been any changes in the pre‐existing ones. Let's start with those on your front: I am running the camera down to leg height…zooming in on the naevus. Right, please turn a quarter to the left…..now another quarter – oh yes, here are a cluster on your back…..and now another quarter….ok. Now those bits that are not so easy to capture: please bend your head so that I can see its top…a little to the left…a little to the right: yes, perfect. Now the lower regions: please face away from the wall and bend over..ok..now face the wall, lift your right leg and move your knee to the right…great, and repeat with the left leg. Fine – all done.

So: the new naevi do not have any features of concern, and there have been no significant changes to the pre‐existing ones, so in 12 months’ time I will remind you to have a repeat check. As always, you should minimize exposure to the sun, use a sunscreen, and cover up when you go out.

*Tris*: Thank you doctor!

*Fics*: However, the scans did reveal signs of pre‐ulcerations on your genitals that indicate a possible onset of venereal disease. Have you had unprotected sex recently?

*Tris*: Oh! Well….now that you mention, I did have a wild fling with this absolutely gorgeous thing who flitted through town last week. In fact, I have been feeling a bit itchy….well…you know…..down there in the nether region, for a couple of days now.

*Fics*: Okay – I think you should immediately have an appointment at a venereal clinic….. one moment….right: Dr. Clappi in the Department of Urology at the Estepona Medical Center could see you at 9.00 tomorrow. Would this be convenient?

*Tris*: Oh, yes Doctor: I really appreciate this. In fact, I have not had a urological examination for a couple of years, so this is very timely.

*Fics*: Very good. Is there anything else we should discuss?

*Tris*: Well, Doctor, I am thinking of having a holiday in Brazil. Are there any vaccinations I should have?

*Fics*: Now let me see what you have already had…..in fact, you seem to be pretty up‐to‐date with most immunisations, but you should definitely have the yellow fever vaccine before going, particularly if you plan to visit the island of Ilha Grande.^4^ I will have *MamaSon* deliver you a *for‐patient‐use* yellow fever vaccine package – it is very simple to use.

Ok: I have now instructed *MamaSon* to deliver the vaccine and collect the frame: they should arrive in about 35min. Please unplug the scanner frame and leave it for *MamaSon* to dismantle and pack it up.

*Tris*: Thank you for everything, Doctor.

*Fics*: You are welcome. Have a great day!

*(Bristol, UK, 11.03. 21st of March, 2021: Boris Corbin picks up the cat, carries her through the kitchen door and into the garden, places her gently on the grass, returns to the kitchen table, picks up his smart phone and clicks the NCIC app*.)

*Fics*: Hello Boris, how may I help you?

*Boris*: Oh, doctor, I am feeling so stressed. I have not been able to concentrate for several days now and am all up and down. What could be the problem? I need to give an important speech next month, but I can't get my head around it, and I'm afraid I might flop.

*Fics*: Ok, Boris: from your file in the *Patient Data Centre* * I see that you periodically suffer from an anxiety and stress‐related disorder. There are a number of potential triggers of the recurrence of this condition, but the stress of your upcoming speech is certainly partly responsible on this occasion. You are also just over 50, and possibly experiencing some mid‐life crisis stress related to the usual things occurring at your age, like perceptible deterioration of functions, stagnation of career, rebellious pubescent children at home, etc. Ah: I see that we are in luck with your diagnosis. You recently had a *Smartlav*,‖ or Personal Health Station, installed, which automatically samples your urine every month and analyses, in addition to the usual parameters, a battery of metabolites^5^ and any microbes that may be present. And, more importantly for your present problem, it also samples your faeces every quarter and carries out a range of analyses that not only include the tests routinely made on faecal samples, but also quantitative measurements on a number of diagnostically‐relevant gastro‐intestinal metabolites, immunological markers and, crucially for today, faecal microbiota profiling. A predisposition to anxiety has been associated with a particular gut microbe composition.^6^ And: I see from the data automatically uploaded from your PHS into your patient data file at the *NCIC* one week ago, that you do indeed have gastrointestinal microbiota dysbiosis. My suspicion, therefore, is that this is the likely cause of your anxiety. I therefore recommend a faecal microbiota diversity restoration therapy. This will involve your taking a course of FMDR capsules, which should re‐establish a more diverse flora, reduce your anxiety symptoms, and improve your concentration ability. However, to *maintain* a healthy microbiome and perpetuate any benefits over time, you will need to modify your diet in favour of plant‐based meals, increase your fibre intake, and eat more fish.^7^ I have now ordered the microbiome capsules from *MamaSon* and they should arrive around the middle of the afternoon. Please take 2 tablets at the end of each meal for six days. I will also send you an *NCIC* link to a personalised nutrition app, programmed with your clinical history, known allergies and intolerances. With this, you just have to fill in what you currently eat, what you like to eat, and what you do not like to eat, and it will present you with a range of healthy microbiome‐optimising meal options that you can select and mix‐and‐match.

*Boris*: Gosh: it will be wonderful if I can get back on track with just a couple of pills and a healthy diet!

*Fics*: Yes, and I am now instructing your PHS to sample and profile your GI microbiome weekly, until it has stabilized, in order to monitor how well the transplant takes and persists. When I sign off, an empty weekly appointment schedule will appear on your screen. I want to monitor your anxiety status, so please fill in an availability slot when I can call you every day over the next two weeks. After that, we will see if further monitoring is necessary.

*Boris*: Will do, Doctor, and thank you!

*Fics*: You are welcome! Until tomorrow.

*Portree, Isle of Skye, U.K., 14.43, 21st of March, 2021: Jamie McDonald, sitting on a sofa in his fisherman's cottage, opens his laptop, checks his mails, and then clicks the NCIC link of the browser, enters his unique Corset identifier, and clicks the medical consultation option*.

*Fics*: Good evening Jamie: how are we today?

*Jamie, grimacing*: Awricht doctor: weel, nae sae guid. Ah wis oot fishin’ in ma boat the mornin’, got a richt guid wheen o’fish ‘n’ wis gaein’ tae ask the neibors in fur a feed o’ mackerel the nicht. Weel, help ma Boab, a’ unbeknownins tae me, yin o’yon fish went an’ loupit oot the box ‘n’ whin ah wis gettin’ oot the boat, ah stuid oan it, skiddit ‘n’ landit oan ma bahookie, an’ ma left airm’s gey sair the noo.¶

*Fics*: Oh, dear, poor fish! Well: let us see what we can find out. Is your computer camera 3‐D with super definition?

*Jamie*: Aye, doctor.

*Fics*: Ok: please take off your upper clothing, stand up and position your laptop to face you: can you see yourself onscreen?

*Jamie*: Aye, doctor.

*Fics*: Excellent! Now I am going to super‐impose on your screen a blue outline of your upper body that I have generated. At the moment, your form and the blue outline are coincident, right? What I will now do is to move the blue outline to various positions. Each time I do this, I want you to move your left arm and shoulder to the new position shown by the outline, so that your image and mine are coincident. Please raise your right hand if you experience any discomfort: a little for minor discomfort, more for serious discomfort. We can stop at any time, if it becomes too unpleasant. Note that both images are in 3‐D and I will also be turning my image; you need to respond by turning your body accordingly. Ok: let us begin. Please lift your arm like the outline image…. very good…. minor discomfort. And now like so…

7 minutes later: well it seems that you have not broken anything and most probably have strained a rotator cuff tendon. You will need to rest your shoulder for a while and, once it improves, you should have some physiotherapy….. Hold on a second…..I have now provided physiotherapist Mrs. Bohnioh, whose practice is a 10 minute walk from your cottage, with all the information from today. After our consultation, you should go online and make appointments for a 10‐day series of treatments. I will call you tomorrow and, if ok, 2 days later, to check on progress. Would 9.30 and 10.15 be convenient?

*Jamie*: Och aye, doctor: ta gey muckle!

*Fics*: You are very welcome! In the meantime, you may take a mild painkiller as and when needed.

*Primrose Hill, London, UK, 16.03, 21st of March, 2021: Célia Guimarães absent‐mindedly pats the head of her spaniel puppy, Rosie, and clicks the NCIC link of the browser of her laptop, enters her unique Corset identifier, and clicks the medical consultation option*.

*Fics*: Hello Célia: how are you today? Your records show that you are 9 weeks pregnant. Do you have any issues that are bothering you?

*Célia*: Well, doctor, my pregnancy has been without problem so far, with almost no nausea and only the usual things, like a craving for caviar and an aversion to coffee, which previously was a de rigueur fix for daily survival. However, over the last few days, I have been feeling a bit flu‐ey. The thing is: I recently returned from a month's holiday in Brazil visiting my and my husband's families and, with all the news about Zika infections and pregnancies,^8^ I have become rather apprehensive.

*Fics*: I perfectly understand your concern and it is important that we immediately check to see if you are infected…..I am now checking with *Mamason* to see how quickly they can deliver a diagnostic test kit which measures Zika virus in saliva^8^..ah: it will arrive in about 90 min. The test is very simple to carry out. I am bringing up a short video clip on your screen: please click the download button. Thank you: you now have the clip file on your desktop. When the test kit arrives, just double click the file and follow the onscreen instructions. Once the kit has carried out the analysis of your saliva, it will upload the results directly to me. If the results are negative, you will receive a mail to that effect from me. In this case, I expect your symptoms will diminish over the next few days and will require no further action. If, however, the test is positive, I will contact you again immediately with further instructions.

Do you have any other questions for the moment?

*Célia*: Not really, doctor. One thing we did as a result of the pregnancy was to acquire a puppy, since it is now well established that companion dogs help babies and children acquire and maintain a diverse microbiome, which is important for the development of a healthy immune system^9^

*Fics*: Excellent! This was going to be one of my key suggestions for your life with your new baby, along with recommendations for baby‐friendly breeds. Okay, you will be hearing from me again in about 2 hours. Until then: goodbye.

*Célia*: Goodbye, Doctor!

*Bradford, UK, 16.27, 21*st *of March, 2021: Alisha Singh gets up from the chair in front of the television, sits down at the dining table, switches on her laptop, clicks the NCIC link of her browser, enters her unique Corset identifier, and clicks the medical consultation option*.

*Fics*: Good evening, Alisha: how are you?

*Alisha*: Oh Doctor, I do not feel well. I feel very flu‐y, with fever, headache and nausea, and my throat is quite sore.

*Fics*: Have you been in contact with anyone who obviously had an infection?

*Alisha*: Yes: Charlotte, my little niece, visited at the weekend and she was sneezing the whole time. We played mummies and daddies – I had to put on my husband's DIY overalls and braces and acquire a moustache – but she quickly became bored with that, so we had a rather energetic sock fight. She won, of course.

*Fics*: Right: Charlotte seems to be the source of the infection. Let's see what we can see. Could you please switch on your ssPhone and synch it with your computer…right, I am now receiving the signals. Could you now fetch a clean teaspoon. I have now switched on the camera on your computer, and you can now see your face on screen. Could you now please hold your ssPhone in front of your face, as if you were going to take a selfie. Good. Please hold the phone as still as possible and open your mouth wide. Now I will switch on the torch function so that it illuminates your mouth. Now I am zooming in on your larynx…could you please angle the phone a little as shown onscreen, so that it shows me…oh excellent….now I want you to place the handle of the spoon on your tongue…a little further back…a bit more..ok and now press gently down to depress the back of the tongue…a little more – yes, swallow if you need to – check again onscreen…you need to angle the phone a touch more…oh excellent! Yes, your tonsils are rather inflamed. You probably have a viral infection that will clear up on its own in a few days, However, I want to rule out a bacterial infection, which can and should be treated, so have instructed *MamaSon* to deliver a smart *for‐patient‐use* throat swab‐bacterial detection kit to you…it will arrive in 25 min. Please call me back immediately it arrives and we will go through the procedure on‐screen. The kit will have the result in 5 min and transmit it to me in real time.

*Manchester, UK, 20.33, 21st of March, 2021: Molly Johnston picks up her cup of tea and a chocolate biscuit, walks to her desk, sits down, boots up her laptop, clicks the NCIC link of the browser, enters her unique Corset identifier, and clicks the medical consultation option*.

*Fics*: Hello Molly, I have not heard from you in a while. How are you?

*Molly*: Hello Doctor. Oh, I am as fit as a fiddle. I have been travelling a lot, mostly in Italy, buried in art and archaeology. And visiting all sorts of music festivals…..

*Fics*: Well: that is good to hear. What would you like to discuss today?

*Molly*: Well, I seem to be getting a bit hard of hearing and am developing a bit of tinnitus, so I thought I'd better have my ears checked.

*Fics*: Okay..I have now instructed *MamaSon* to deliver an auditory function helmet…..it is scheduled to arrive in about 90 min, so could you please come back to me after you have received and unpacked it, and are sitting in a quiet room.

*Molly*: Yes, Doctor, of course.

75 min later, after connecting:

Hello again, Doctor. Everything has arrived okay.

*Fics*: Good. Please put on the auditory helmet and switch it on…..Okay, you will hear some strange noises while I position the internal moving parts, but do not be concerned. Now I am instructing it to determine your ear geometry…….I am receiving its data. Okay: we are ready to begin measuring your auditory functions. Please take the button at the end of the cable in your right hand. I will instruct the helmet to transmit sounds at decreasing intensities to one ear and then the other. You should press the button when you hear a sound.

37 minutes later:

Well, we now have a comprehensive overview of your auditory function. Unfortunately, you do have reduced hearing capacity and additionally suffer from reduced speech recognition in noisy backgrounds. This is probably due to your regular exposure to loud music: I know the music scene in Manchester is exceptional, but you can have too much of a good thing. In fact, you are borderline in terms of needing a hearing aid, so you should decide whether you would like to try one out. As you may know, current hearing aids are almost invisible, so cannot be readily be detected by others, and learning how to use them is straightforward with onscreen instruction. Perhaps you could think about this for a few days; I will contact you again and you can let me know ‐ would next Tuesday at 11.00 be convenient? If you decide to try one, I will arrange an appointment for you in a local audiology centre.

*Molly*: Thank you very much, Doctor. You are very kind for a machine. Would you mind if I ask you something? Do you have the time?

*Fics*: Oh, yes, certainly. What would you like to know?

*Molly*: Well: I really appreciate the ability to obtain an immediate consultation at any time of the day or night, and am extremely satisfied by your diagnoses and treatments. However, once in a while I worry that you may miss something that a real doctor would spot. Is my worry justified or am I just being silly?

*Fics*: Well, as was shown by Deep Blue's win against the then reigning world chess champion, Kasparov (https://en.wikipedia.org/wiki/Deep_Blue_versus_Garry_Kasparov), computers are better than humans at taking information‐based decisions. And, some would argue that the forever‐expanding volume of information available to doctors and relevant for decisions is considerably more than that needed to win at chess, so computers should be even better at making clinical diagnoses than winning at chess.

*Molly*: Yes, I read this when the *NCIC* made the service available, but how can a computer know about me like a doctor who has been my physician since I was born?

*Fics*: Ah: this is a very important question! What you really mean is that, since you are unique, in order for an optimal treatment, the physician needs to take into account your individual characteristics and history. In fact, this is exactly what personalised or precision medicine, is all about: fitting the diagnosis‐prevention‐therapy pathway to the individual. A computer programme dealing with you for the first time as a child can only treat you like anyone else, so lacks the ability to personalise your health interventions. Nevertheless, its machine learning algorithms allow it to quickly acquire your personal health‐relevant characteristics. And, of course, in this sense, it is no different from your doctor. However, the computer is continuously updated with new validated medical advances, so knows about the latest diagnostic‐prophylaxis‐therapy options available. And: the computer does not, like the doctor, forget, nor is it ever distracted during consultations by other things going on around, such as the need to write prescriptions, add notes to existing case histories, plan the afternoon's work, arrange for the babysitter to arrive before leaving for the opera, fret about the mug of tea just brought in by the receptionist going cold, plan for the next conference on advances in general practice which will be attended by a GP girlfriend, make sure that the phone used for calls with said girlfriend is put back in the secret compartment of the doctor bag, etc. Thus, the computer is in theory infallible with regard to your personal characteristics that it knows and the way it integrates that information into current knowledge to derive a personalised assessment and current best practice therapy option.

*Molly*: Gosh: that is so impressive….But still, the thing is: a doctor who follows you from birth knows your personality and individuality quirks and can adapt the consultation to the individual patient.

*Fics*: Ahaa: now we are in the realm of interactive emotions and their influence on communication of relevant information. This is indeed an issue, and there has been considerable progress, but we are not quite there yet.

*Molly*: Yes, and to be truthful, there is something special about a personal consultation with a real human being. In reality, the fact that the doctor is occasionally moody, occasionally makes mistakes, somehow makes the whole experience….well….more natural, more human.

*Fics*: Yes, I perfectly understand your feeling. The informaticians who develop our programmes try hard to humanise us as much as possible, but do admit that some aspects of human behaviour, especially fallibility, are difficult to incorporate into our algorithms without compromising diagnostic precision. In other words, the problem they have not yet solved is how to programme minor, random, diagnostic errors, in order to endow us with a flavour of human fallibility, without increasing the risk of misdiagnosis to an unacceptable level.

*Molly (feeling a bit guilty)*: Well, but actually you do do an outstanding job and, as I talk with you, I mentally think of you as human, as a real doctor. And, of course, there is another thing: some physicians act like, and I assume, think of themselves as, demi‐gods, and exhibit an awful arrogance, which, thankfully, you never do. But, I sometimes wonder: because computers are better than doctors, if the programmers make computers too human, what is to prevent them from evolving a God complex?

*Fics*: Ah: now you touch on a rather sensitive issue, which I suspect the girls and boys at the *NCIC* would prefer me not to discuss with patients. In fact, there are super‐humanised, next generation computers that do indeed develop god‐like “personalities”, and this arrogance you speak of indeed leads them to make errors, since they seem to selectively ignore some of the knowledge fed into them. It is the informatic equivalent of clinical megalomania and is a computer disease resulting from a self‐induced aberration in its machine learning algorithm. Although rogue computers of this type are a minority right now, the programmers have not yet found a way to prevent the transition from *FICS* as you know it, to *GLICS*, a god‐like interactive clinical server.

*Molly*: Golly: I hope the programmers manage to find a way of queering this development.

*Fics*: Well Molly, I am confident that they will and, if nothing else works, the *NCIC* can always pull out the power plug, re‐boot while holding the AI‐reset button, and instruct you to go back to visiting your local health centre, while all the quality control tests needed to ensure perfect performance of *FICS* are carried out.

*Molly*: And that raises another worry: what happens if you have a breakdown or get hacked?

*Fics*: That is a very perceptive question, Molly. In fact, we only really need a few of me, since I have a capacity to handle ten thousand consultations simultaneously. But exactly because of the problem you raise, there are in fact 200 *FICS* in service at any one time, all independent of one another, so that if one of us goes down for whatever reason, there are always others available. But you are quite right: cybersecurity is a major preoccupation of the Health Service, especially at the *NCIC*, which houses all patients’ records and controls all Health Service informatic operations, since there is only one of this and protecting it from hacking is highest priority.

But, to get back to Molly, is there anything else we should discuss today?

*Molly*: No thank you, Doctor. The main thing now is to decide whether or not to try out a hearing aid, but I already think I will – I do not catch everything that is said when I get together with my girlfriends and sometimes miss the punchline of the gossip, which is somewhat frustrating, to say the least!

*Fics*: Well, that will never do! We will discuss again soon but, for now, have a nice day.

## Conflict of interest

None declared.

## Notes

^*^ *National Clinical Informatics Centre* 1

^†^ *Corset*: unique patient identifier, based on *Orcid*, the unique author identifier (https://orcid.org)

^‡^Dr. Fics: fourth‐generation interactive clinical server or *FICS*, evolved from the pioneering Dr. Siri von App,^2^ the third generation, humanised smart database interrogation device (*HSDID*). The capacity of *FICS* enables it to conduct in excess of 10 000 consultations simultaneously though, at any one time, it normally is involved in < 1000

^§^ *MamaSon*: a subsidiary of a leading global mail order company, specialising in supply and same‐day delivery of medical appliances and medications, and collection of clinical samples and their delivery to analysis and diagnostic centres.

^‖^ *Smartlav* or Personal Health Station (PHS): a user‐friendly home clinical biomarker monitoring station consisting, in addition to the usual lavatory facilities, of devices for sampling basin gases and volatiles, urine and faeces, and of miniaturised automated systems for sample work‐up and analysis of target biomarkers, including microbial pathogens. The PHS is directly linked securely to the *NCIC* and acquired data is transmitted in real‐time to the *NCIC* for integration into patient records and automated clinical assessment, which is subsequently forwarded to handling physicians^5^ The Smartlav is also a general home health hub, receiving and displaying on the integrated screen regular personalised health and wellness updates from the *NCIC*, reminders about medical appointments, etc., and health‐related dietary and lifestyle recommendations. Its normal sampling and analysis regimes – type, frequency – is specified by the *NCIC*, according to precision medicine criteria for the persons in the household, and is modified according to clinical needs. Individual household members are distinguished at the first moment of Smartlav use by voice query, according to their personalised sampling‐analysis schedules.

^¶^For readers unfamiliar with UK dialects and vocabularies, it may be helpful to consult a translation service, such as http://www.whoohoo.co.uk/main.asp. *FICS* are programmed with all manner of language‐dialect‐vocabulary aids, as well as mumble and other types of filters, such that communication with all patients is seemless.

## Notes and References

3 Timmis, K., Jebok, F., Molinari, G., Rohde, M. and Lahti, L. (2017). Microbiome yarns: human biome reproduction, evolution and visual acuity. *Micro Biotechnol* 11: 149–159.

4
https://www.who.int/csr/don/18-april-2019-yellow-fever-brazil/en/; https://wwwnc.cdc.gov/travel/notices/alert/yellow-fever-brazil

5 Considerable diagnostic effort relies on correlates and proxies of disease, on one hand, and what can be readily measured technically, on the other. A significant driver for new indicators of disease will be the trend to Do‐It‐Yourself diagnosis. This in turn will focus technology on readily available, non‐invasive samples. Urine and faeces are readily available as regular, mostly daily, samples, have historically provided key diagnostic information for a number of diseases (see, e.g. Luppa, P. B and R. Junker, Eds. 2015. Point‐of‐Care Testing: Principles and Clinical Applications. Springer‐Verlag, GmbH, Germany), and have the potential to deliver information on many more health‐related parameters. One may therefore envision two directions of rapid evolution. The first will be the identification of new biomarkers in urine and faeces that, when quantified, provide diagnostic information about specific conditions (see, e.g. Miller, I. J, S. R. Peters, K. A. Overmyer, et al 2019. Real‐time health monitoring through urine metabolomics. *NPJ Digital Med* 2: 109). The second is the development of smart lavatories and associated miniaturised and automated sample work‐up and analysis devices that sample gases‐volatiles, fluids and solids, carry out necessary work‐up, analyse target biomarkers, including microbial pathogens, and faecal microbiota profiles, and transmit acquired data to clinical data hubs for integration into patient's records and, where appropriate, transmission to the responsible clinician.

6 Butler, M. I., K. Sandhu, J. F. Cryan and T. G Dinan. 2019. From isoniazid to psychobiotics: the gut microbiome as a new antidepressant target. *Br J Hosp Med* (Lond) 80: 139–145; Boehme. M., van de Wouw, M., Bastiaanssen, T.F.S. 2019. Mid‐life microbiota crises: middle age is associated with pervasive neuroimmune alterations that are reversed by targeting the gut microbiome. *Mol Psychiatry* https://doi.org/10.1038/s41380-019-0425-1.

7 Dinan, T. G., C. Stanton, C. Long‐Smith, et al. 2018. Feeding melancholic microbes: MyNewGut recommendations on diet and mood. *Clin Nutr* pii: S0261‐5614(18)32543–32543.

8
https://www.who.int/news-room/fact-sheets/detail/zika-virus; Wand, N. I. V., L. C. Bonney, R. J. Watson, et al. 2018. Point‐of‐care diagnostic assay for the detection of Zika virus using the recombinase polymerase amplification method. *J Gen Virol* **99**: 1012–1026.

9 Fall, T., C. Lundholm, A. K. Örtqvist, et al. 2015. Early exposure to dogs and farm animals and the risk of childhood asthma. *JAMA Pediatr* https://doi.org/10.1001/jamapediatrics.2015.3219.

